# Assessing the impact of a comprehensive mental health program on frontline health service workers

**DOI:** 10.1371/journal.pone.0294414

**Published:** 2023-11-21

**Authors:** Emily J. Ward, Maren S. Fragala, Charles E. Birse, Matt Hawrilenko, Casey Smolka, Geetu Ambwani, Millard Brown, John H. Krystal, Philip R. Corlett, Adam Chekroud

**Affiliations:** 1 Spring Health, New York, NY, United States of America; 2 Quest Diagnostics, Secaucus, NJ, United States of America; 3 Department of Psychiatry, Yale School of Medicine, New Haven, CT, United States of America; University of Udine: Universita degli Studi di Udine, ITALY

## Abstract

Mental health issues are a growing concern in the workplace, linked to negative outcomes including reduced productivity, increased absenteeism, and increased turnover. Employer-sponsored mental health benefits that are accessible and proactive may help address these concerns. The aim of this retrospective cohort study was to evaluate the impact of a digital mental health benefit (Spring Health) on frontline healthcare service workers’ clinical and workplace outcomes. The benefit was sponsored by a national health services company from 2021–2022 and included mental health screening, care navigation, psychotherapy and/or medication management. We hypothesized program use would be associated with improvements in depression and anxiety symptoms, and increased productivity and retention. Participants were employees enrolled in the benefit program, had at least moderate anxiety or depression, at least 1 treatment appointment, and at least 2 outcome assessments. Clinical improvement measures were PHQ-9 scale (range, 0–27) for depression and GAD-7 scale (range, 0–21) for anxiety; workplace measures were employee retention and the Sheehan Disability Scale (SDS) for functional impairment. A total of 686 participants were included. Participants using the mental health benefit had a 5.60 point (95% CI, 4.40–6.79, *d* = 1.28) reduction in depression and a 5.48 point (95% CI, 3.88–7.08, *d* = 1.64) reduction in anxiety across 6 months. 69.9% (95% CI, 61.8%–78.1%) of participants reliably improved (≥5 point change) and 84.1% (95% CI, 78.2%–90.1%) achieved reliable improvement or recovery (<10 points). Participants reported 0.70 (95% CI, 0.26–1.14) fewer workdays per week impacted by mental health issues, corresponding to $3,491 (95% CI, $1305–$5677) salary savings at approximately federal median wage ($50,000). Furthermore, employees using the benefit were retained at 1.58 (95% CI, 1.4–1.76) times the rate of those who did not. Overall, this evaluation suggests that accessible, proactive, and comprehensive mental health benefits for frontline health services workers can lead to positive clinical and workplace outcomes.

## Introduction

Mental health disorders are the leading cause of disability worldwide [1] and an estimated half of adults with a mental illness go untreated each year [2]. This highlights a critical gap in our healthcare system and a pressing need to address the barriers to mental health care. Healthcare and frontline workers are especially vulnerable to mental health issues, due to higher levels of stress and trauma in their work [3]. Additionally, those in healthcare may feel more stigma associated with mental health issues [4]. More recently, stressors such as the COVID-19 pandemic can have adverse mental health impacts on healthcare workers including elevated risk of stress, burnout, moral injury, depression, and trauma [5] and highlighted a need for the development of flexible and accessible mental health solutions for healthcare workers [6, 7]. Increasing access and effectiveness of mental health care is essential to ensuring the well-being of these crucial members of our society.

More generally, mental health issues can have a significant impact on workplace outcomes, where such issues have been linked to negative outcomes such as reduced productivity [8, 9], increased absenteeism [10], increased turnover [8, 9]. Employees with untreated mental health issues may struggle to manage their workload effectively, leading to decreased productivity, and they may also be more likely to miss work. Furthermore, untreated mental health issues can lead to burnout [11, 12], and thus higher turnover. Addressing mental health concerns is therefore critical to improve both employee well-being and the performance of the organization.

An employer-sponsored, evidence-based mental health benefit is an imperative strategy for addressing mental health concerns in the workplace. A common means through which employees receive mental health benefits are Employee Assistance Programs (EAPs) or behavioral health benefits through a medical plan. EAPs typically offer employees confidential resources to assist with personal or work issues, including mental health issues. However, EAPs have shown their low utilization rates, lack of effectiveness [13] and limited clinical improvement [14, 15]. As such, employers may need to consider new strategies, such as offering comprehensive and proactive mental health benefits.

In healthcare, a myriad of mental health services have emerged including digital tools designed specifically to support the mental health of hospital-based health care workers [7], blended care combining digital and person-to-person mental health support services [6], and even a publicly funded doctors’ mental health programing in Australia [12]. More generally, mental health benefits that are accessible, proactive, and comprehensive have begun to show promise improving employee mental health and benefiting the performance of the organization [16]. Such benefits offer short wait times for appointments, flexible treatment plans and service offerings, including a variety of treatment from coaching to psychotherapy to medication management. The most comprehensive benefits offer various evidence-based elements, such as a digital platform for mental health screening, online cognitive behavioral therapy resources, free or low-cost access to care, and a symptom tracking framework is in place to facilitate measurement-based care. In particular, they often provide guidance for employees [16, 17], such as using care navigators or concierges to assist in choosing the right treatment option and therapists. Such guidance is typically lacking in traditional EAPs. Previous research has demonstrated that one such mental health program that incorporates these elements delivered both positive mental health benefits to employees and financial outcomes to employers by improving depression and anxiety symptoms and increasing workplace engagement [18].

In this evaluation, our aim was to evaluate the impact of this comprehensive mental health program on healthcare service workers’ (1) clinical outcomes, specifically depression and anxiety) and (2) workplace outcomes, specifically productivity and retention. We hypothesized that the program would be associated with improvements in depression and anxiety symptoms, and increased worker productivity and retention, from which we could estimate financial savings due to the program.

## Methods

The Yale Institutional Review Board approved the study (IRB protocol ID: 2000029276) and determined that it was not research involving human subjects. Informed consent was not required because the data were anonymized prior to analysis. The study followed the Strengthening the Reporting of Observational Studies in Epidemiology (STROBE) reporting guideline for observational studies. Information that could identify individuals during and after data collection was accessible to some of the authors, but all analyses were conducted without personally identifying information.

### Program design

We used data from an employer-sponsored digital mental health benefit (Spring Health; Spring Care Inc). The program incorporates several evidence-based components to increase access and utilization of mental healthcare, such as telephone and video appointments with care navigators who help individuals find appropriate care, online cognitive behavioral therapy resources, and free or low-cost access to psychotherapy and medication management through video or in-person sessions. Participants could schedule unlimited calls with their care navigators.

After completing the initial assessment (see Questionnaires), individuals received a personalized care plan and could schedule appointments with care navigators, therapists, medication managers, or coaches. If a participant’s care team deemed them in need of more intensive services, they were referred to an external online mental health benefit program by their clinicians. All care navigators were licensed mental health clinicians with a master’s degree level or above, all therapists had a master’s or doctoral-level license, and all medication managers were medical doctors or doctors of osteopathic medicine. All clinicians had at least 3 years of experience post-supervision before joining the network.

To lower the financial barriers to accessing care, participants could book an unlimited number of free appointments with their care navigator. Additionally, they could schedule appointments with a program therapist and/or medication manager. Participants had access to 6 covered employer-sponsored sessions per year. Additional sessions with the same providers could be continued with copays and deductibles according to behavioral health plan selection and coverage.

### Questionnaires

The program includes a digital mental health assessment tool, which enables a proactive symptom monitoring system to support measurement-based care. Within the digital platform, individuals electively completed a series of questionnaires to identify common mental health difficulties (such as stress, anxiety, sleeping, eating, or relationship issues). In this evaluation, participants completed the Patient Health Questionnaire 9-item scale (PHQ-9) [19, 20] for depression and a form of the Sheehan Disability Scale (SDS) [21] for functional impairment, along with additional self-report questionnaires based on the problems identified by the participant. For those with anxiety, this included the Generalized Anxiety Disorder 7-item scale (GAD-7) [22]. They were also asked how optimistic they feel that therapy can help them and chose regular intervals to complete follow-up assessments, with the default being every 2 weeks.

### Inclusion criteria

Employees were health services professionals, specifically, laboratory and diagnostic services who were eligible for the benefit between January 1, 2022 and December 31, 2022. Enrollment was free to help eliminate financial barriers to care. Employees were included in the current evaluation if they were over 18, located in the U.S., were employed for a minimum of 15 days (determined using eligibility files). Employees were counted as in treatment if they had at least 1 therapy or medication management appointment from the mental health benefit during the evaluation time period. This group was compared against the other employees in the retention analysis. Participants did not receive monetary reward.

For the clinical and workplace outcomes analyses, individuals must have also completed assessments at least two or more times (including the PHQ-9, GAD-7 or SDS): an initial assessment and an additional assessment taken after the start of therapy. First, the initial assessment must have been completed in the month prior to the start of therapy to serve as a baseline. Second, at least one additional assessment(s) must have been completed at least a month after the start of therapy and assessments taken up to 6 months after the last therapy appointment were included. Participants for whom these data were missing were excluded prior to analysis.

To be included in the clinical outcomes analyses, participants must have had a baseline PHQ-9 or GAD-7 score above clinical cutoff points (PHQ-9 ≥10 or GAD-7 ≥10) [19, 20, 22, 23]. The final sample was determined by the total number of participants who met these criteria.

### Measures

#### Depression and anxiety

*Depression symptoms* were measured with the PHQ-9 (range, 0–27), consisting of nine items assessing the frequency of a range of depression symptoms. *Anxiety symptoms* were measured with the GAD-7 (range, 0–21), consisting of seven items assessing the frequency of a range of anxiety symptoms. Both PHQ-9 and GAD-7 items are scored 0–3 (*not at all* to *nearly every day*). PHQ-9 and GAD-7 scores were outcome variables for assessing symptom change (continuous outcomes), reliable improvement [24] (5-point decrease in the PHQ-9 [25] and 4-point decrease in the GAD-7), and reliable improvement with recovery (both reliable improvement and ending in the subclinical range [20], corresponding to a score <10). Depression and anxiety symptoms were modeled independently rather than as comorbid factors. Response to prior treatment, treatment resistance, or current psychotropic medication usage was not measured.

#### Factors associated with clinical improvement

The primary outcome was change in clinical symptoms over *time* (measured from the start of therapy [t = 0] and estimated at the end of 6 months [coded as t = 1]). The initial assessment measured *treatment optimism* (self-report, range 1–10). Demographics were collected when individuals enrolled in the program: *age*, *race* (optional; classified into two broad categories: white and person of color) and *gender* (optional; classified as female, male, or other).

#### Workplace measures

*Time back in the workplace* (self-report, 0–7) was measured with an abbreviated Sheehan Disability Scale (SDS) [21], a self-report measure of family, work, and social functional impairment due to emotional symptoms. Specifically, participants indicated how many days they missed work and were unproductive in the last week due to their mental health symptoms. These two values were added together for analysis. The estimated changes in missed days and unproductive days per week were used to calculate the savings achieved in six months using the following formula: (changes in absenteeism + changes in unproductive days) × number of work weeks in 6 months × daily salary. *Employee retention* (retained/departed) was determined from employee eligibility files and an employee was categorized as departed when they did not appear on the files for 30 days. Retention rates were compared between those employees who engaged with the mental health program and those who did not.

### Statistical analyses

We used multiple regression models to estimate clinical and workplace outcomes. The regression models accommodated different types of outcomes through linking functions: an identity link for continuous outcomes (depression, anxiety), a Poisson model with a log link for count data (number of days impacted by mental health issues), and a logit link for categorical outcomes (employee retention, reliable change or recovery). To address repeated observations for the same participants (such as scores across assessments), we used a mixed-effects model with repeated observations nested within *participant* as a random intercept.

Symptom change was modeled using a quadratic polynomial with time since start of treatment as the main effect of interest. To determine which factors were associated with symptom change, the set of measures described above (optimism about treatment, age, gender, race) were included as covariates. To control for unmeasured effects in approach or skill, *provider* was included as a random slope. Improvement rates were transformed to total improvement from baseline to treatment endpoint using the delta method. The delta method multiplies the desired duration to be quantified (i.e., 6 months from the start of treatment) by the rate of change given by linear and quadratic coefficients to obtain standard errors from a function that combines model parameters. Reliable improvement or recovery were predicted separately using logistic models with time since start of treatment as the main effect of interest. Regression coefficients from these models were converted to probabilities to estimate the percentage of participants who had reliable improvement in or recovery from depression and anxiety symptoms. Time back in the workplace was modeled using a Poisson regression with time since the start of treatment as the main effect of interest and a random slope for participant. Cohen *d* effect sizes for clinical and workplace improvement were calculated by dividing the overall effect size by the baseline SD with established thresholds to categorize effects as small (*d* <0.50), medium (*d* <0.8), and large (*d* >0.8).

All statistical tests were 2-sided with statistical significance set at α levels of .05. All analyses were conducted in R, version 4.2.1 [26].

## Results

### Participant characteristics

**Fig 1:**
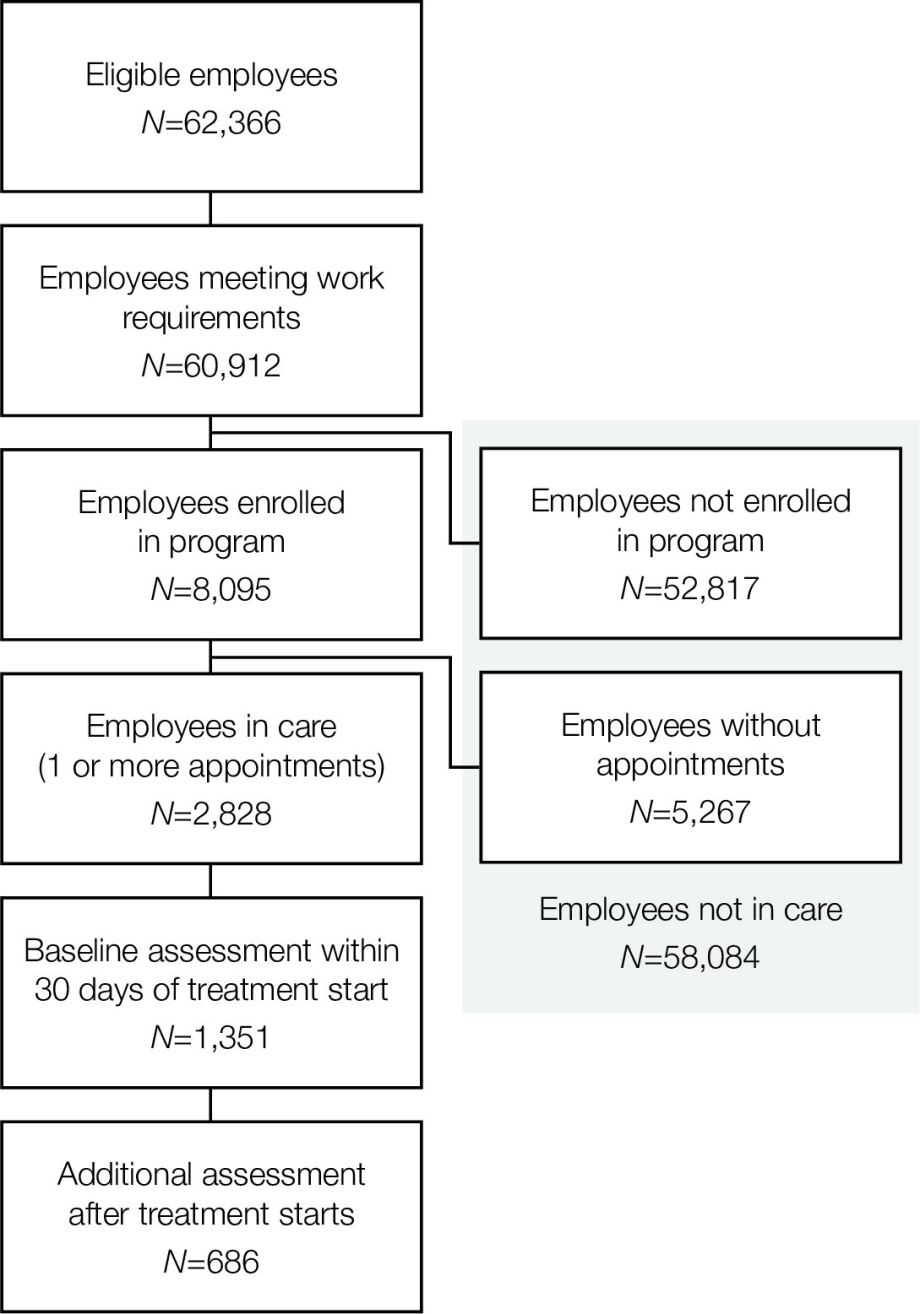
Participant flow chart, showing progression of inclusion in the current evaluation. Participants were considered *in care* with the mental health benefit program if they had 1 or more appointments during the evaluation period. Those who were not enrolled with the program or were enrolled but did not have any appointments were considered *not in care*. For modeling clinical and workplace outcomes, participants must have met additional criteria including having a baseline assessment and follow-up assessment after treatment started.

62,366 employees were eligible for the benefit (Fig 1) and a total of 60,912 employees met the employment criteria. 8,095 employees (13.3%) enrolled in the program and 2,828 (35%) had at least 1 therapy or medication management appointment from the mental health benefit during the evaluation time period. 58,084 employees did not engage in care. 1,351 participants also completed an initial assessment and an additional assessment (including the PHQ-9, GAD-7 or SDS) taken after the start of therapy, 686 of whom completed the additional assessment(s) at least a month after the start of therapy and within 6 months after the last therapy appointment.

Among the 686 participants who met the inclusion criteria (Table 1), the age at enrollment ranged from 19–70 years (mean = 40.7, SD = 11.9). 532 of 684 [77.6%] who reported gender information were female; and 329 of 633 [48.0%] who reported demographic information were white. Mean optimism for treatment was 7.0 (SD = 2.1) out of 10 points. 305 participants screened positive (scores ≥10 points) [19, 20, 22, 23] for depression or anxiety, (although not all provided race, gender, or treatment optimism ratings). 80 screening positive for only depression, and 38 screening positive for only anxiety, and 187 screening positive for both depression and anxiety, corresponding to a 61.3% comorbidity rate, similar to the general population [27–29]. Among these participants, baseline scores were moderate to severe for both PHQ-9 (mean [SD], 16.0 [4.4]) and GAD-7 (mean [SD], 14.9 [3.3]). For the workplace outcomes, 324 participants had at least two assessments that included SDS scores.

**Table 1:** Baseline sociodemographic characteristics and clinical overview.

	Overall (N = 686)
**age**	
mean (SD)	40.7 (11.9)
median [min, max]	39.0 [19.0, 70.0]
**gender**	
female	532 (77.6%)
male	145 (21.1%)
non-binary	2 (0.3%)
other	3 (0.4%)
Missing	4 (0.6%)
**race & ethnicity**	
asian	39 (5.7%)
black	151 (22.0%)
latinx/hispanic	85 (12.4%)
middle eastern	1 (0.1%)
mixed-race	14 (2.0%)
native american	2 (0.3%)
other	11 (1.6%)
person of color ‐ general	1 (0.1%)
prefer not to answer	20 (2.9%)
white	329 (48.0%)
Missing	33 (4.8%)
**days in treatment**	
mean (SD)	96.8 (85.1)
median [min, max]	70.2 [0.0217, 335]
**attended therapy session**	
no	30 (4.4%)
yes	656 (95.6%)
**attended med. mgmt. session**	
no	561 (81.8%)
yes	125 (18.2%)
**total therapy sessions | yes**	
mean (SD)	4.08 (3.77)
median [min, max]	3.00 [1.00, 32.0]
**total med mgmt sessions | yes**	
mean (SD)	1.91 (1.42)
median [min, max]	1.00 [1.00, 8.00]
**PHQ9 at baseline**	
mean (SD)	11.2 (6.32)
median [min, max]	10.0 [2.40, 27.0]
missing	191 (27.8%)
**GAD7 at baseline**	
mean (SD)	9.70 (5.53)
median [min, max]	9.00 [1.48, 21.0]
missing	194 (28.3%)
**Screened positive (≥10pts)**	
both PHQ & GAD	187 (27.3%)
GAD only	38 (5.5%)
PHQ only	80 (11.7%)
missing	381 (55.5%)
**PHQ9 at baseline | positive**	
mean (SD)	16.0 (4.38)
median [min, max]	15.0 [10.0, 27.0]
missing	419 (61.1%)
**GAD7 at baseline | positive**	
mean (SD)	14.9 (3.33)
median [min, max]	15.0 [10.0, 21.0]
missing	461 (67.2%)

### Overall clinical outcomes

95.6% (656 of 686) participants used psychotherapy, attending 4.1 (SD = 3.8) therapy sessions on average and spending approximately 3 months in treatment (96.8 [85.1] days). 18.2% (125 of 686) attended at least one medication management appointment. Among those who attended medication appointments, the mean (SD) number of appointments was 1.9 (1.4). The median times to first available appointment during the evaluation period were 1.1 days (IQR, 1.0–1.9 days) for psychotherapy and 1.1 days (IQR, 1.0–1.4 days) for medication management.

**Fig 2:**
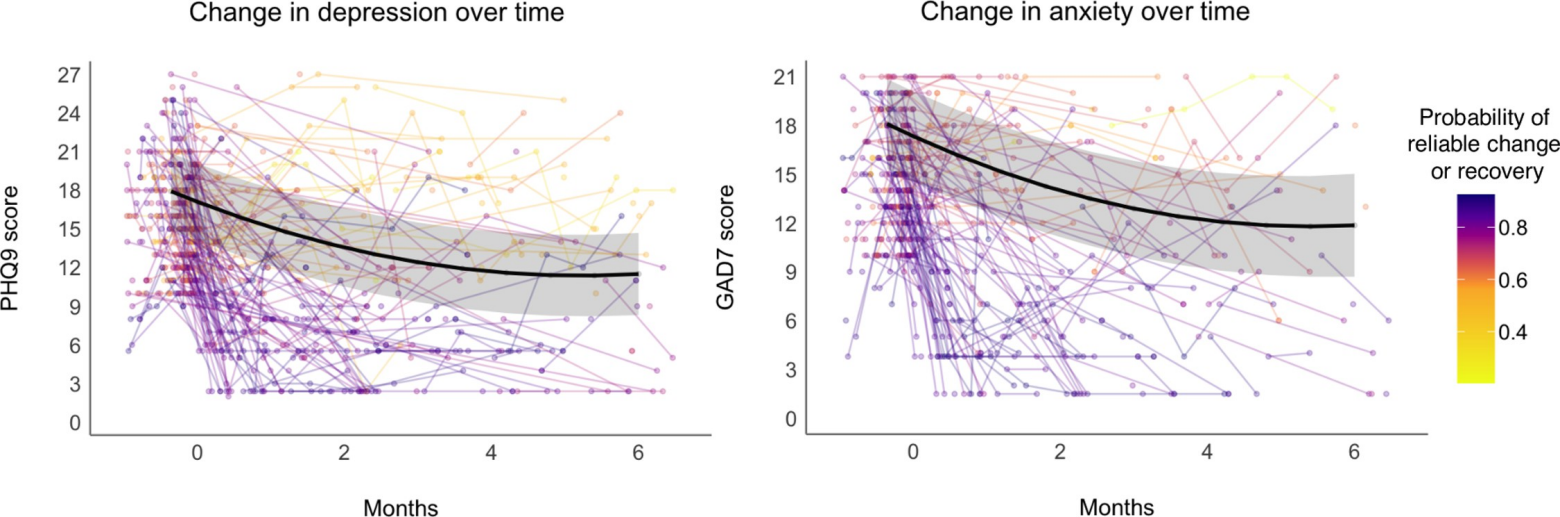
Change in depression and anxiety across the 6-month treatment period. Overall improvement (black; 95% CI) is shown with individual participant trajectories, colored according to the probability of each achieving reliable change or recovery within 6 months.

Overall, PHQ-9 scores decreased across a 6-month treatment period (*b*_*tim*e_ = -13.16, *b*_*time2*_ = 7.56; *Ps* < .001) (Table 2 and Fig 2A), resulting in a total reduction of 5.60 points (95% CI, -6.79 to -4.40) over treatment, corresponding to a large effect size (*d* = -1.28; 95% CI, -1.55 to -1.00). GAD-7 scores also decreased across a 6-month treatment period, (*b*_*time*_ = -12.31, *b*_*time2*_ = 6.83; *Ps *< .001) (Table 2 and Fig 2B), resulting in a total reduction of 5.48 points (95% CI, -7.08 to -3.88), corresponding to a large effect size (*d* = -1.64; 95% CI, -2.12 to -1.16). There were no significant effects of age, race, gender, or optimism for treatment (Table 2).

**Table 2:** Multiple regression results for clinical outcomes (depression and anxiety).

	Depression (PHQ-9 scores)			Anxiety (GAD-7 scores)		
*Predictors*	*Estimates*	*std*. *Error*	*p*	*Estimates*	*std*. *Error*	*p*
(Intercept)	17.10	1.52	**<0.001**	17.33	1.41	**<0.001**
time (6m)	-13.16	1.15	**<0.001**	-12.31	1.38	**<0.001**
time (6m)^2^	7.56	0.91	**<0.001**	6.83	1.20	**<0.001**
race [people of color]	-0.64	1.03	0.539	-1.67	1.01	0.100
race [white]	-1.07	0.99	0.279	-1.05	0.99	0.289
gender [male]	-0.12	0.67	0.862	-0.17	0.65	0.793
gender [not specified]	3.26	3.36	0.333	-4.19	2.60	0.108
gender [other]	3.01	2.84	0.291	-0.97	3.35	0.773
age	-0.02	0.02	0.375	-0.04	0.02	0.099
optimism	-0.21	0.12	0.089	-0.19	0.12	0.126
**Random Effects**						
σ^2^	18.69			15.94		
τ_00_	7.74 _member_id_			4.86 _member_id_		
τ_11_	13.05 _member_id.time_6m_			18.47 _member_id.time_6m_		
	0.46 _provider_id.time_6m_			6.88 _provider_id.time_6m_		
ρ_01_	1.00 _member_id_			1.00 _member_id_		
ICC	0.48					
N	258 _member_id_			217 _member_id_		
	191 _provider_id_			168 _provider_id_		
Observations	773			592		
Marginal R^2^ / Conditional R^2^	0.158 / 0.559			0.275 / NA		

Results of mixed-effects logistic regression models (Table 3) indicated that 69.9% (95% CI, 61.8%–78.1%) of participants’ symptoms reliably improved and 84.1% (95% CI, 78.2%–90.1%) achieved reliable improvement or recovery.

**Table 3:** Multiple regression results for reliable improvement and recovery (depression and anxiety).

	Depression (PHQ-9)						Anxiety (GAD-7)					
	Reliable Change			Recovery			Reliable Change			Recovery		
*Predictors*	*Odds Ratios*	*CI*	*p*	*Odds Ratios*	*CI*	*p*	*Odds Ratios*	*CI*	*p*	*Odds Ratios*	*CI*	*p*
(Intercept)	0.22	0.16–0.31	**<0.001**	0.17	0.12–0.25	**<0.001**	0.22	0.15–0.32	**<0.001**	0.19	0.13–0.28	**<0.001**
time (6m)	8.23	4.88–13.89	**<0.001**	7.69	4.47–13.25	**<0.001**	11.74	5.81–23.73	**<0.001**	9.83	5.00–19.31	**<0.001**
**Random Effects**												
σ^2^	3.29			3.29			3.29			3.29		
τ_00_	1.41 _member_id_			1.87 _member_id_			1.35 _member_id_			1.30 _member_id_		
ICC	0.30			0.36			0.29			0.28		
N	263 _member_id_			263 _member_id_			222 _member_id_			222 _member_id_		
Observations	781			781			599			599		
Marginal R^2^ / Conditional R^2^	0.155 / 0.409			0.136 / 0.449			0.180 / 0.418			0.160 / 0.398		

### Workplace outcomes

Participants enrolled in care with the mental health program had 1.58 (OR = 1.58, 95% CI, 1.43–1.76) (Table 4) greater odds of being retained than participants who were not enrolled. Turnover rate for those in care was 15.0% compared to 21.8% for those who were not, corresponding to a 31.2% relative reduction in employee turnover.

**Table 4:** Logistic regression results for employee retention.

	Retention		
*Predictors*	*Odds Ratios*	*CI*	*p*
(Intercept)	3.58	3.51–3.65	**<0.001**
In Care	1.58	1.43–1.76	**<0.001**
Observations	60912		
R^2^ Tjur	0.001		

Among those in care, participants reported that mental health issues impacted their work resulting in a total SDS score of 3.29 (3.53) days per week affected, consisting of 1.56 (2.02) days missed and 1.73 (1.95) days unproductive due to these issues. Overall, the incidence of mental health issues that impacted work decreased across a 6-month treatment period (IRR = 0.61, 95% CI, 0.41–0.90; *P* = .012) (Table 5), resulting in a total of 0.70 (95% CI, 0.26–1.14; *d* = 0.20; 95% CI, 0.07–0.32) workdays per week recovered over treatment.

**Table 5:** Regression results for days impacted by mental health issues.

	SDS (days missed + days unproductive)		
*Predictors*	*Incidence Rate Ratios*	*std*. *Error*	*p*
(Intercept)	1.77	0.13	**<0.001**
time (6m)	0.61	0.12	**0.012**
**Random Effects**			
σ^2^	0.46		
τ_00 member_id_	1.08		
τ_11 member_id.time_6m_	0.38		
ρ_01 member_id_	0.74		
ICC	0.74		
N _member_id_	324		
Observations	659		
Marginal R^2^ / Conditional R^2^	0.017 / 0.740		

The recovery of 0.70 workdays per week corresponded to a six-month salary savings for an employee at the federal median wage (approximately $50,000) was $3,491(95% CI, $1,305–$5,677) per employee per 6 months, and ranged from $1,053 for employees at the federal minimum wage to $13,964 for employees making at least $200,000 per year (Table 6).

**Table 6:** Estimated salary savings from increased time in workplace during a 6-month period, in US dollars.

Annual Salary ($)	Salary savings in 6 mo ($, 95% CI)
15080	1053 (393, 1712)
25000	1745 (652, 2839)
50000	3491 (1305, 5677)
63179	4411 (1649, 7174)
75000	5236 (1957, 8516)
100000	6982 (2609, 11355)
125000	8727 (3262, 14193)
150000	10473 (3914, 17032)
200000	13964 (5219, 22709)

## Discussion

The aim of evaluation was to determine the impact of using a comprehensive digital mental health benefit (Spring Health) on frontline healthcare service workers’ clinical and workplace outcomes. Consistent with our hypothesis, we found that the program was associated with significant reductions in depression and anxiety symptoms, corresponding to 35% absolute decrease in depression severity and 37% absolute decrease in anxiety severity. Although our evaluation is an observational cohort study, these effects are more than double the reduction of 16% in depression severity that has been shown to occur spontaneously without treatment [30]. In addition, nearly 70% of all participants saw reliable improvement in their symptoms and over half achieved reliable improvement or recovery (i.e., scores that dropped into the subclinical range). These effect sizes are on par with outcomes obtained from this mental health program in other employee populations [18], and are now shown for another population that is especially vulnerable to mental health issues, due to higher levels of stress and trauma in healthcare work.

We also found that the mental health program was associated with positive workplace outcomes, including recovering 0.70 workdays per week through improved productivity and fewer missed days. This productivity improvement is over twice what is expected spontaneously (∼0.45 days recovered) [31]. These days back in the workplace correspond to a six-month salary savings of about $3500 for employees at approximately the federal median wage. Additionally, we found that those engaged in care with the mental health program had 1.6 times the odds of being retained compared to those who did not, consistent with previous research of the program’s effect on retention [18].

These results show the effectiveness of a novel intervention in a real-world setting. Such evidence complements insight from clinical trials by including factors that would impact effectiveness in the real world [32]. The high quality and strong positive outcomes observed in both the previous and the current evaluation may be due to the accessible and proactive nature of the program. Over 13% of all eligible employees enrolled in the program, showing the potential for strong utilization. Among those who enrolled, we saw high use of psychotherapy, with nearly 35% having at least 1 therapy appointment during the evaluation period. This suggests the program increases access (e.g., through free or low-cost access to psychotherapy and medication management). Furthermore, utilization of the program was similar between white employees and employees of color, where 48% of participants were white and 43% were people of color (the remainder did not specify their race) and when included as a covariate, race–broadly explored between white and non-white participants–did not significantly impact any of the results. The program also proactively supports individuals who have not yet reached clinical thresholds for mental health issues, since the majority of employees (55.5%) who enrolled in the program did not screen positive for either depression or anxiety. Among those who do screen positive for depression or anxiety (10 points or more on PHQ-9 or GAD-7), the results suggest that the program proactively finds and promotes effective treatment methods (e.g., through consultations with care navigators to assist members in finding appropriate care and through regular assessments and symptom tracking to facilitate measurement-based care).

The results of this evaluation have important implications for both employee mental health and workplace performance. Given the high levels of stress faced by frontline healthcare workers, they face elevated risk for mental health issues [3] and the COVID-19 pandemic highlighted the importance of prioritizing and protecting the mental health and well-being of the healthcare workforce through self-care strategies, and evidence-based interventions [5]. But they also require mental health solutions that accommodate demanding and unpredictable workdays. Compared to traditional Employee Assistance Programs (EAPs) with low utilization rates, lack of effectiveness [13] and limited clinical improvement [14, 15], the comprehensive program includes higher engagement, regular check-in assessments coupled with proactive outreach from Care Navigators if conditions do not improve as expected, and ongoing outreach campaigns to members and non-enrolled employees. By providing comprehensive and accessible mental health benefits, including therapy and medication management, employers can help mitigate the negative impact of these demands on employee well-being and workplace outcomes. These results also complement findings in occupational medicine more broadly, in which workplace health initiatives, including mental health, can offer significant benefits to the entire system, by enhancing employees’ quality of life and work [33] and providing return on investment for employers [34], such as through reduced total expenditures and improved healthcare utilization patterns (lower emergency room visits, more mental health visits) [35].

Overall, this evaluation provides important evidence for the potential clinical and workplace benefits of employer-sponsored mental health programs for frontline healthcare workers. By prioritizing mental health in the workplace, employers can improve both employee wellbeing and workplace outcomes, contributing to a healthier and more productive workplace.

### Strengths and limitations

This evaluation has several strengths, such as a diverse and real-world sample of health services employees, with 43% of the participants being people of color. The participants had a baseline comorbidity rates similar to the general population [28, 29] and the magnitude of improvement is greater than what is observed to occur spontaneously [30, 31]. However, as an observational cohort study that only examined outcomes before and after engaging in the program, it cannot determine a causal link between improvements and engagement in the program. Furthermore, the evaluation was conducted in a single healthcare service organization and we did not break down effects by employee role. As such, these effects may not be generalizable to other industries or populations. In particular, the evaluation period was during the COVID-19 pandemic, which was especially stressful for frontline workers [3]. No spatial or temporal data regarding COVID-19 outbreak waves was modeled in this evaluation. Although we controlled for race broadly in this evaluation, there may be differences between specific racial groups or comorbidities that could affect engagement and outcomes. Finally, this evaluation was limited to depression and anxiety, but these are but a snapshot of the mental health problems suffered by health services workers, which range from symptoms to disorders (mood, anxiety, personality, cognitive, substance abuse, psychotic).

## Conclusions

This retrospective cohort study suggests that employer-sponsored mental health benefits to employees can lead to positive clinical and workplace outcomes. Employees who used the mental health benefit reported significant reductions in depression and anxiety symptoms, and improvements in productivity, and absenteeism. Furthermore, they were retained at a higher rate than those employees who did not. Providing proactive, comprehensive, and evidence-based mental health benefits may ultimately benefit employers through increased productivity and reduced turnover.

## Supporting information

S1 Checklist STROBE statement—checklist of items that should be included in reports of observational studies. (PDF)
